# Regulation of connective tissue growth factor expression by miR-133b for the treatment of renal interstitial fibrosis in aged mice with unilateral ureteral obstruction

**DOI:** 10.1186/s13287-021-02210-2

**Published:** 2021-03-10

**Authors:** Dan Cao, Yuan Wang, Yingjie Zhang, Yinping Zhang, Qi Huang, Zhong Yin, Guangyan Cai, Xiangmei Chen, Xuefeng Sun

**Affiliations:** Department of Nephrology, Chinese PLA General Hospital, Chinese PLA Institute of Nephrology, State Key Laboratory of Kidney Diseases, National Clinical Research Center for Kidney Diseases, 28 Fuxing Road, Beijing, China

**Keywords:** miR-133b, Target gene, Aged, Renal interstitial fibrosis

## Abstract

**Introduction:**

Renal interstitial fibrosis, an important pathological feature of kidney aging and chronic renal failure, is regulated by mesenchymal stem cells (MSCs). We have previously demonstrated low expression of miR-133b in MSC-derived extracellular vesicles (MSC-EVs) in aged rats. However, miR-133b can mediate the inhibition of epithelial-mesenchymal transition (EMT) of renal tubules induced by transforming growth factor-β1 (TGF-β1). We investigated the effect of miR-133b for the treatment of geriatric renal interstitial fibrosis and evaluated its target genes.

**Methods:**

We performed real-time polymerase chain reaction to detect miR-133b expression induced during EMT of HK2 cells by TGF-β1 at different concentrations (0, 6, 8, and 10 ng/mL) and at different time points (0, 24, 48, and 72 h). The target genes of miR-133b were validated using the dual-luciferase reporter assay. In vitro experiments were performed to evaluate mRNA and protein expression of miR-133b targets, E-cadherin, α-smooth muscle actin (SMA), fibronectin, and collagen 3A1 (Col3A1), in HK2 cells transfected with miR-133b under TGF-β1 stimulation. A 24-month-old unilateral ureteral obstruction (UUO) mouse model was established and injected with transfection reagent and miR-133b into the caudal vein. The target gene of miR-133b and other parameters mentioned above such as mRNA and protein expression levels and renal interstitial fibrosis were detected at 7 and 14 days.

**Results:**

miR-133b expression gradually decreased with an increase in TGF-β1 concentration and treatment time, and the miR-133b mimic downregulated connective tissue growth factor (CTGF) expression. The dual-luciferase reporter assay confirmed CTGF as a direct target of miR-133b. Transfection of the miR-133b mimic inhibited TGF-β1-induced EMT of HK2 cells; this effect was reversed by CTGF overexpression. miRNA-133b expression significantly increased (approximately 70–100 times) in mouse kidney tissues after injection of the miRNA-133b overexpression complex, which significantly alleviated renal interstitial fibrosis in mice with UUO.

**Conclusion:**

miR-133b exerted targeted inhibitory effects on CTGF expression, which consequently reduced TGF-β1-induced EMT of HK2 cells and renal interstitial fibrosis in aged mice with UUO.

**Supplementary Information:**

The online version contains supplementary material available at 10.1186/s13287-021-02210-2.

## Background

Aging is characterized by significant changes in the structure and function of the kidney, even in the absence of age-related comorbidities. Renal interstitial fibrosis is an important pathological feature of kidney aging [1], and bone marrow mesenchymal stem cells (MSCs) play an important role in the regulation of renal interstitial fibrosis. Transplantation of the bone marrow from young mice into aged mice was shown to significantly reduce renal fibrosis as well as the expression of markers associated with aging in the recipient mice. However, bone marrow cells did not directly replace parenchymal cells, but instead exerted paracrine effects on renal parenchymal cells [2]. Further, the injection of MSCs and MSC-derived extracellular vesicles (MSC-EVs) was shown to significantly alleviate renal interstitial fibrosis in a mouse model of unilateral ureteral obstruction (UUO) [3, 4].

Aging can significantly alter the number of stem cells and their regenerative capacity and functions [5]. Our previous research revealed significant differences in the expression profiles of microRNAs (miRNAs) of MSC-EVs derived from the bone marrow of young and aged rats. We found that miR-133b-3p was underexpressed in MSC-EVs of aged rats; however, miR-133b could inhibit the epithelial-mesenchymal transition (EMT) of renal tubular cells induced by transforming growth factor (TGF)-β1 [6]. The inhibitory effects of MSC-EVs against renal fibrosis decreased with age. miR-133b of MSC-EVs derived from aged rats has been suggested to exhibit important intervening effects on renal fibrosis [7].

miR-133b ameliorates cardiac fibrosis [8–10], reduces TGF-β1-mediated EMT of bladder smooth muscle epithelial cells [11], and directly targets the connective tissue growth factor (CTGF) [9, 11]. Whether CTGF is a target involved in miR-133b-mediated inhibition of EMT of renal tubular epithelial cells remains unclear, and studies are warranted to investigate the effect of exogenous expression miR-133b in geriatric renal interstitial fibrosis.

In this study, EMT of the human renal proximal tubular epithelial cell line HK2 was stimulated by TGF-β1 in vitro to investigate the role of miR-133b and its target genes in this process. A mouse model of UUO was established in aged C5BL/6J7 mice (aged 24 months), which were then intravenously injected with a miR-133b transfection complex to verify the effect of miR-133b overexpression in geriatric renal fibrosis.

## Methods

### TGF-β1 stimulation and miR-133b transfection

The HK2 cells were cultured in the Dulbecco’s modified Eagle medium (DMEM)/F12 (Corning, USA) supplemented with 5% fetal bovine serum. After reaching 50% confluency, the cells were synchronized in serum-free DMEM/F12 for 18 h and then stimulated with TGF-β1 at 6, 8, and 10 ng/mL concentrations for 24, 48, and 72 h. miR-133b mimic and miRNA mimic control (GenePharma, China) were transfected into HK2 cells for 6 h using the jetPRIME® transfection reagent according to the manufacturer’s instructions (Polyplus-transfection, France). Following transfection, the cells were cultured in DMEM/F12 with 5% serum for 18 h and then incubated with DMEM/F12 with 5% serum and 8 ng/mL of TGF-β1 (PeproTech, USA) for 48 h.

### RNA extraction and real-time polymerase chain reaction (RT-PCR)

Total RNA was extracted from HK2 cells and kidney tissues of each group using Trizol and subsequently used to synthesize miR-133b and U6 cDNA using the miScript II RT Kit (QIAGEN, China). Primers specific for target genes were designed with reference to their mRNA-coding regions in GenBank using the Primer 5.0 software. The primer sequences were verified using BLAST. Total RNA was used to synthesize cDNA of target genes using the ReverTra Ace qPCR RT Master Mix kit (TOYOBO, Japan). The expression of genes encoding miR-133b, CTGF, E-cadherin, α-smooth muscle actin (SMA), fibronectin, collagen 3A 1 (Col3A1), U6, and glyceraldehyde 3-phosphate dehydrogenase (GAPDH) was detected on the ABI-prism-7500 sequence detection system (Applied Biosystems, USA) using the miScript SYBR Green PCR Kit (QIAGEN). The relative expression levels were calculated using *U6* or *GAPDH* as internal control.

### Western blot analysis

After lysis and denaturation of HK2 cells or kidney tissues from each group using the radioimmunoprecipitation assay (RIPA) buffer, proteins (50 μg) were separated by 8% sodium dodecyl sulfate-polyacrylamide gel electrophoresis (SDS-PAGE) and then transferred onto nitrocellulose (NC) membranes. After blocking with 1x casein for 1 h to prevent non-specific binding, NC membranes were incubated with the following primary antibodies overnight at 4 °C: (a) rabbit monoclonal E-cadherin antibody (BD Bioscience, USA) diluted 1:100, (b) rabbit monoclonal anti-α-SMA antibody (Abcam, UK) diluted 1:300, (c) mouse monoclonal anti-CTGF antibody (Abcam) diluted 1:200, (d) rabbit polyclonal anti-fibronectin antibody (Proteintech, USA) diluted 1:500, (e) rabbit polyclonal Col3A1 antibody (Proteintech) diluted 1:500, and (f) mouse monoclonal β-actin antibody (Beyotime, China) diluted 1:10,000. The membranes were washed with TBST (Tris-buffered saline with Tween-20, 20 mM of Tris, 140 mM of NaCl, and 0.1% Tween-20) and then probed with 1:1000 diluted secondary antibodies at room temperature (25 °C) for 2 h. Enhanced chemiluminescence (ECL) western blotting kit (APPLYGEN, China) was used to detect the target bands, and β-actin was used as an internal reference to calculate the relative expression levels of proteins in each experimental group.

### Prediction of target genes of miR-133b

The target genes of miR-133b were predicted using three commonly employed target gene prediction software, namely TargetScan (http://www.targetscan.org/), miRBase (http://www.mirbase.org/), and PicTar (https://pictar.mdc-berlin.de/).

### Dual-luciferase reporter assay

The seed sequence for the binding of CTGF and miR-133b was searched using the bioinformatics software TargetScan. The sequence 5′-AUUUGUUGAGUGUGACCAAAA-3′ containing the 3′-untranslated region (UTR) of CTGF was synthesized and cloned into a luciferase reporter vector GP-miRGLO (GenePharma) and termed as miRGLO-Wt-CTGF. A mutant sequence 5′-AUUUGUUGAGUGUUGGAUUAA-3′ of the target was also synthesized and cloned into the plasmid to obtain miRGLO-Mut-CTGF, which was used as the negative control.

293T cells from the logarithmic growth phase were lysed with pancreatin and seeded in 48-well plates for 24 h. After reaching 80% confluency, the cells were transfected using the cell fusion reagent. The synthesized miR-133b mimic and miRNA mimic control (NC-miR) were co-transfected with miRGLO-Wt-CTGF or miRGLO-Mut-CTGF, respectively, into 293T cells using the jetPRIME® transfection reagent according to the manufacturer’s instructions. After 48 h, the cells were lysed using a passive lysis buffer (Promega, USA) and the cell lysate was collected. The luciferase activity of the lysate was detected according to the instructions indicated in the dual-luciferase reporter assay system kit (Promega).

### Immunofluorescence staining

HK2 cells were seeded at a density of approximately 10^5^ cells/well in 6-well plates with sterile glass coverslips which were disinfected by autoclaving. The plates were placed in an incubator at 37 °C with 5% carbon dioxide for 6 h to allow the cells to adhere to the glass cover slips. After synchronization and transfection as mentioned above, the cells were incubated with DMEM/F12 containing 5% serum with or without 8 ng/mL of TGF-β1 for 48 h. The cells were then fixed with 4% paraformaldehyde at room temperature for 20 min and treated with 0.2% Triton X-100 for 2 min for permeabilization. The cells were then blocked with 5% bovine serum albumin (BSA) at room temperature for 1 h and then treated with anti-E-cadherin rabbit monoclonal primary antibody (1:100 dilution), α-SMA antibody (1:100 dilution), rabbit polyclonal Col3A1 antibody (1:100 dilution), fibronectin antibody (1:100 dilution), and mouse monoclonal anti-CTGF antibody (1:100 dilution) diluted in 5% BSA at 4 °C overnight. After washing with phosphate-buffered saline (PBS), the cells were probed with an anti-rabbit fluorescein isothiocyanate (FITC)-conjugated fluorescent secondary antibody (Beyotime) (1:400 dilution) at room temperature (25 °C) in the dark for 2 h. The slides were then washed with PBS and treated with 4′,6-diamidino-2-phenylindole (DAPI) (ZSGB-BIO, China) fluorescence nuclear staining mounting medium. The expression of α-SMA and E-cadherin in HK2 cells from each group was observed under a fluorescence microscope (× 100) with random fields of vision.

### Experimental animals and establishment of the UUO model

Animal care and experiments were performed according to the guidelines of the Institutional Animal Care and Use Committee of Chinese PLA General Hospital. A total of 28 female C5BL/6J7 mice (SPF grade), aged 24 months, weighing 20 ± 2 g, were provided by SPF Biotechnology Co., Ltd. (Beijing, China). Mice were randomly divided into sham (*n* = 8), UUO+NC-miR-133b (*n* = 10), and UUO+miR-133b (*n* = 10) groups.

To establish the UUO model, each mouse was anesthetized by an intraperitoneal injection of pentobarbital (50 mg/kg) and the abdominal cavity was opened under sterile conditions. The left ureter was dissociated, double-ligated, and disconnected at 15 mm below the renal pelvis with a 4-0 thread. The abdominal cavity was closed by layered suture. In the control group, the abdominal cavity was closed immediately after the ureter was dissociated.

The miR-133b mimic or NC-miR (3 mg/kg) was diluted to 1 μg/μL concentration using endotoxin-free purified water. The transfection reagent Entranster-in vivo (Engreen, China) was diluted in a 10% glucose solution to a final glucose concentration of 5%. The transfection complex was prepared by mixing the two agents and by incubating for 15 min. The UUO+miR-133b and UUO+NC-miR groups were administered with miR-133b and the NC-miRNA transfection complex (100 μL/animal), respectively, by caudal vein injection at 24 h before surgery and once every 3 days thereafter. The sham group was administered with the same volume of normal saline by caudal vein injection. The mice were euthanized at 7 and 14 days after the UUO procedure (four mice from the sham group and five mice from the UUO group were euthanized at each time point). The kidney tissues were collected from the obstructed side for western blotting, real-time PCR, and pathological analyses.

### Pathological examination of the kidney tissue

The kidney tissue was fixed in 10% neutral formaldehyde, dehydrated with ethanol, embedded in paraffin, and cut into 2-μm sections. Morphological changes in the kidney tissue were observed by periodic acid-Schiff (PAS) and Masson’s trichrome staining. After staining with the Masson’s trichrome, 10 fields of vision were selected under a light microscope (× 400). The area of each field of vision and area of collagen fibers stained in green were measured using the Image-Pro Plus software. The relative area of collagen deposition was calculated as follows: area of collagen fibers stained in green/area of field of vision × 100%.

### Statistical analysis

The data were analyzed using the SPSS 17.0 software, and the results were expressed as mean ± standard error of mean (SEM). The differences among the experimental groups were analyzed using one-way analysis of variance (ANOVA) with a completely random design. Results with *P* < 0.05 were considered statistically significant.

## Results

### Inhibitory effect of TGF-β1 on miR-133b

**Fig. 1:**
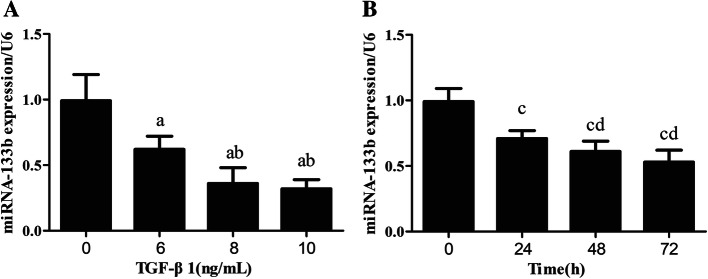
Expression of miR-133b in HK2 cells stimulated with different concentrations of TGF-β1 and at different time points. The experiment was performed in triplicate, and the expression of miR-133b was detected by real-time PCR. **A** The expression of miR-133b in HK2 cells stimulated with different concentrations of TGF-β1. a: *P* < 0.05 versus 0 ng/mL of TGF-β1 group; b: *P* < 0.05 versus 6 ng/mL of TGF-β1 group. **B** The expression of miR-133b in HK2 cells stimulated with 8 ng/mL of TGF-β1 at different time points. c: *P* < 0.05 versus the 0–h group; d: *P* < 0.05 versus the 24-h group

The real-time PCR results of miR-133b expression analysis showed that the stimulation of HK2 cells with TGF-β1 at concentrations of 0, 6, 8, and 10 ng/mL for 48 h resulted in a gradual decrease in the expression of miR-133b in a TGF-β1 concentration-dependent manner. However, no significant difference was observed between the 8-ng/mL- and 10-ng/mL-treated groups (Fig. 1A). The expression of miR-133b after stimulation of HK2 cells with 8 ng/mL of TGF-β1 for 0, 24, 48, and 72 h gradually decreased upon increasing stimulation periods; no significant difference was observed between the 48-h and 72-h treatment groups (Fig. 1B).

### Effect of miR-133b on the morphology of TGF-β1-stimulated HK2 cells

**Fig. 2:**
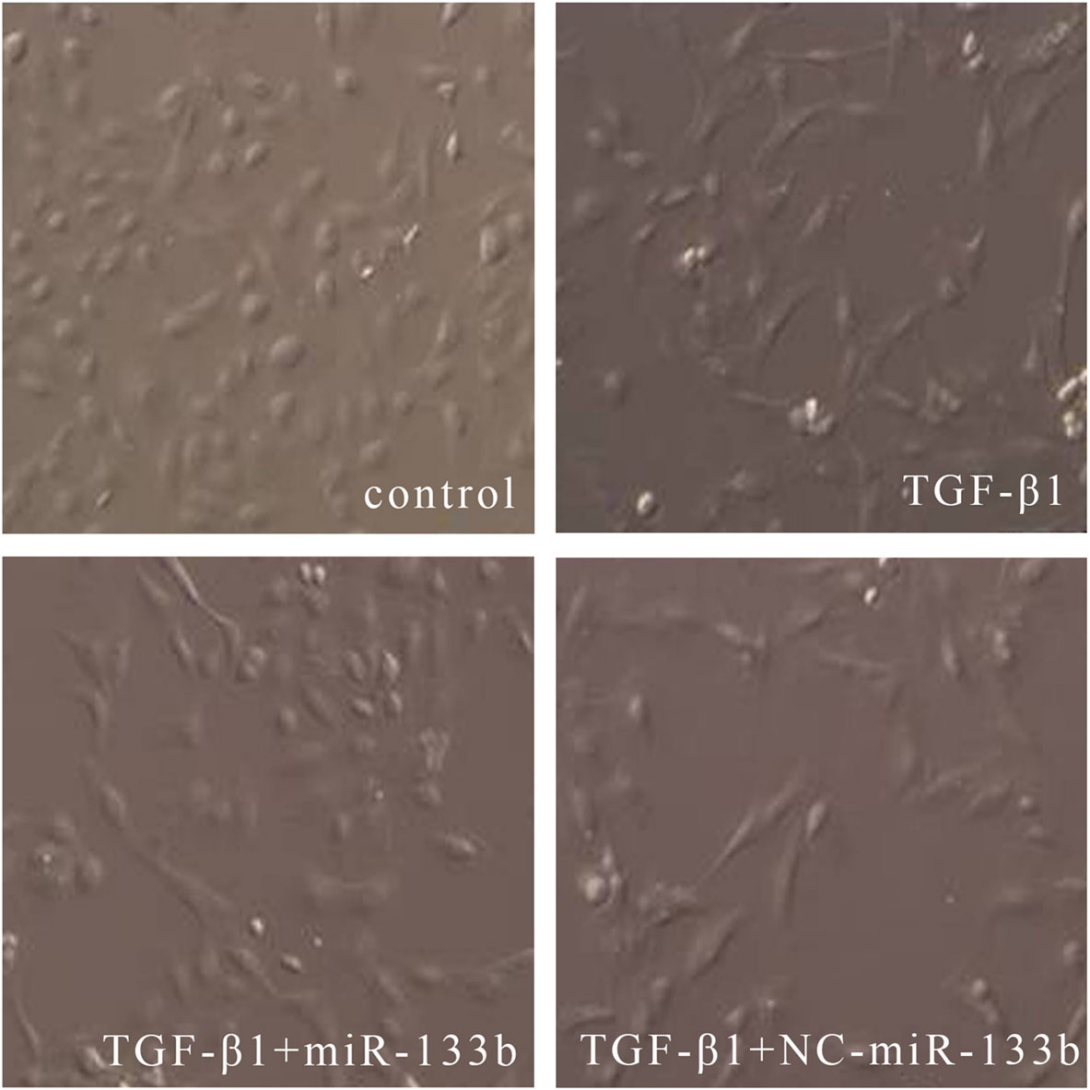
Morphological changes in HK2 cells following different stimulation conditions. The morphological changes in cells were observed under an inverted microscope (× 100). Control: HK2 cells were cultured for 48 h without stimulation; TGF-β1: HK2 cells were stimulated with 8 ng/mL of TGF-β1 for 48 h; TGF-β1+miR-133b: HK2 cells transfected with miR-133b were stimulated with 8 ng/mL of TGF-β1 for 48 h; TGF-β1+NC-miR: HK2 cells transfected with NC-miR were stimulated with 8 ng/mL of TGF-β1 for 48 h. HK2 cells exhibited a round or an oval shape. After stimulation with 8 ng/mL of TGF-β1 for 48 h, the cells became slender and fusiform, and their intercellular space significantly widened. While most cells transfected with miR-133b continued to maintain the morphology of epithelial cells, the NC-miR-transfected cells exhibited the morphology of fibroblasts

Morphological changes in the cells were observed under an inverted microscope. HK2 cells exhibited a round or an oval shape and were arranged in a cobblestone pavement-like pattern. After stimulation with 8 ng/mL of TGF-β1 for 48 h, the cells became slender and fusiform, and their intercellular space significantly widened. While most cells transfected with miR-133b mimic continued to maintain the morphology of epithelial cells, the NC-miR-transfected cells showed the morphology of fibroblasts (Fig. 2).

### *CTGF* is a direct target gene of miR-133b

We used three different miRNA target gene prediction software (TargetScan, PicTar, and miRbase) and found *CTGF* to be a potential target of miR-133b. The target site was located between 1027 and 1033 bp of the 3′-UTR of *CTGF*. The results of DAVID bioinformatics resources suggested that *CTGF* might be involved in TGF-β-mediated fibrosis and in other signaling pathways.

**Fig. 3:**
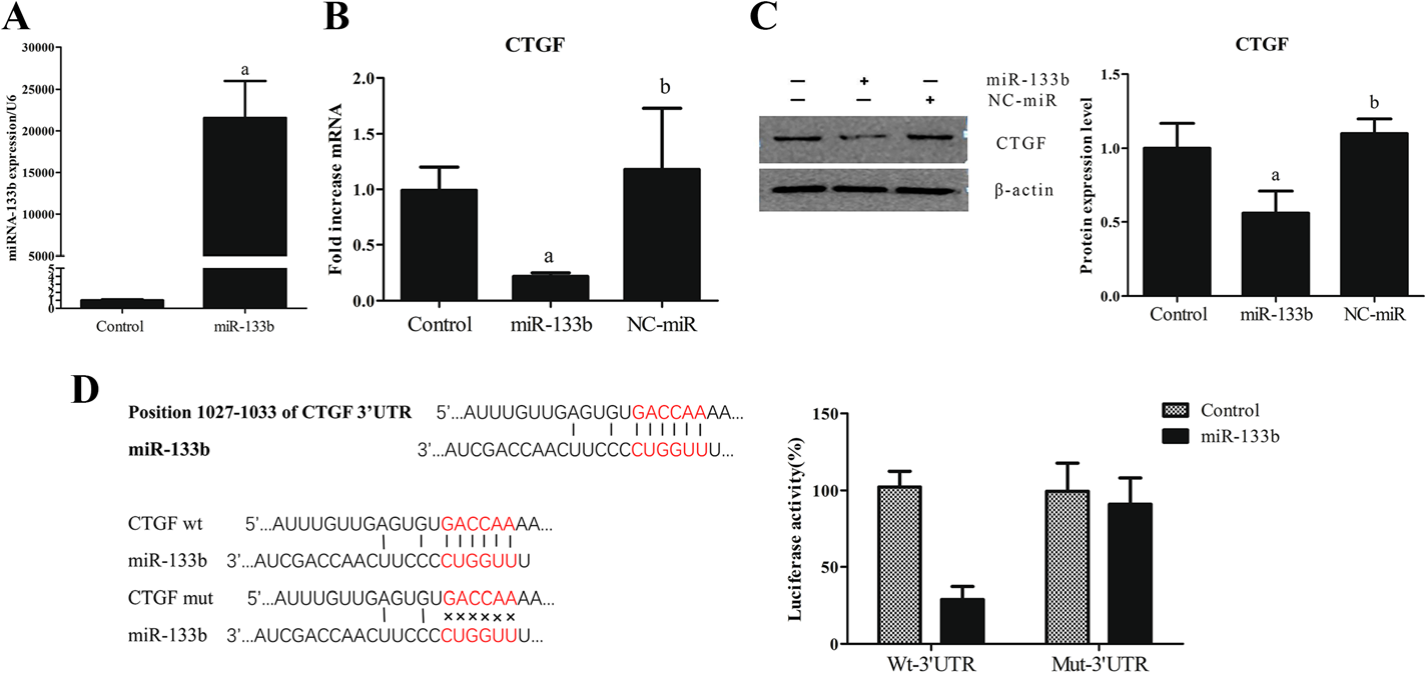
*CTGF* is a direct target gene of miR-133b. Transient transfection of miR-133b mimic into HK2 cells for 48 h. **A** miR-133b expression was detected by real-time PCR. **B** *CTGF* expression was detected by real-time PCR. **C** CTGF expression was analyzed by western blotting. **D** Dual-luciferase reporter assay confirmed *CTGF* as a direct target gene of miR-133b

After transient transfection of a miR-133b mimic into HK2 cells for 48 h, we performed real-time PCR and western blotting and found a significant increase in the miR-133b expression (increased by approximately 21,566 times) (Fig. 3A), which confirmed the successful transfection of miR-133b. While the expression of CTGF mRNA and protein significantly decreased following miR-133b transfection, there was no change in the group transfected with NC-miR (Fig. 3B, C).

### miR-133b inhibits EMT of HK2 cells induced by TGF-β1

**Fig. 4:**
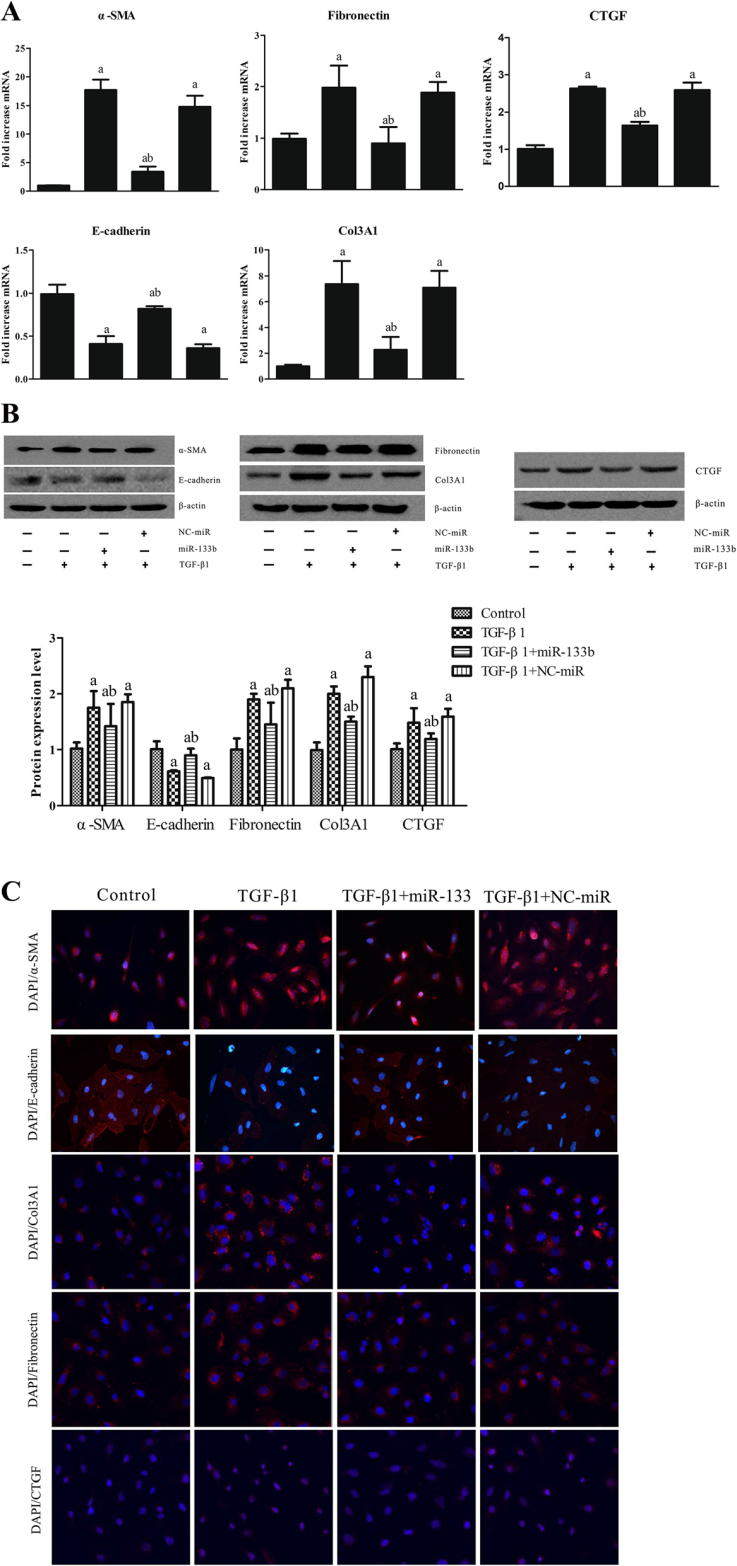
miR-133b inhibited EMT of HK2 cells induced by TGF-β1. Normal (untransfected) HK2 cells or HK2 cells transfected with miR-133b mimic/NC-miR for 24 h were stimulated with 8 ng/mL of TGF-β1 for 48 h. **A** Real-time PCR results. **B** Western blotting results. **C** Cell immunofluorescence assay results. a: *P* < 0.05 versus the control group; b: *P* < 0.05 versus the TGF-β1 group

Normal HK2 (untransfected) cells or HK2 cells transfected with miR-133b mimic/NC-miR for 24 h were stimulated with 8 ng/mL of TGF-β1 for 48 h. The results of real-time PCR and western blotting analyses showed that miR-133b significantly inhibited the downregulated mRNA and protein expression of E-cadherin as well as the upregulated mRNA and protein expression of α-SMA, fibronectin, Col3A1, and CTGF induced by TGF-β1 (Fig. 4A, B).

Immunofluorescence results showed that the fluorescence intensity of α-SMA, Col3A1, fibronectin, and CTGF increased and that of E-cadherin decreased in cells treated with TGF-β1 as compared to that in control cells. Compared to cells stimulated with TGF-β1 alone, the cells treated with TGF-β1 and miR-133b showed a decrease in the fluorescence intensity of α-SMA, Col3A1, fibronectin, and CTGF and an increase in the intensity of E-cadherin. Further, no significant change was observed in the fluorescence intensity of α-SMA, Col3A1, E-cadherin, fibronectin, and CTGF between the cells treated with TGF-β1 and NC-miR-133b and those treated with TGF-β1 alone (Table 1; Fig. 4C).

**Table 1:** Comparison of fluorescence intensity of α-SMA and E-cadherin in HK2 cells under different stimulation conditions

Group	Number of experiments	Average fluorescence intensity of cells	
		α-SMA	E-cadherin
Control	3	0.021 ± 0.003	0.175 ± 0.028
TGF-β1	3	0.103 ± 0.043*	0.013 ± 0.001*
TGF-β1+miR-133b	3	0.041 ± 0.005*^,#^	0.067 ± 0.009*^,#^
TGF-β1+NC-miR	3	0.092 ± 0.004*	0.009 ± 0.001*

The immunofluorescence experiment was performed in triplicate, and five fields of vision were selected for each group. The fluorescence intensity of cells was observed under a confocal microscope (× 100); *P < 0.05 versus the control group; ^#^*P* < 0.05 versus the TGF-β1 group

### Overexpression of CTGF reverses the protective effect of miR-133b

**Fig. 5:**
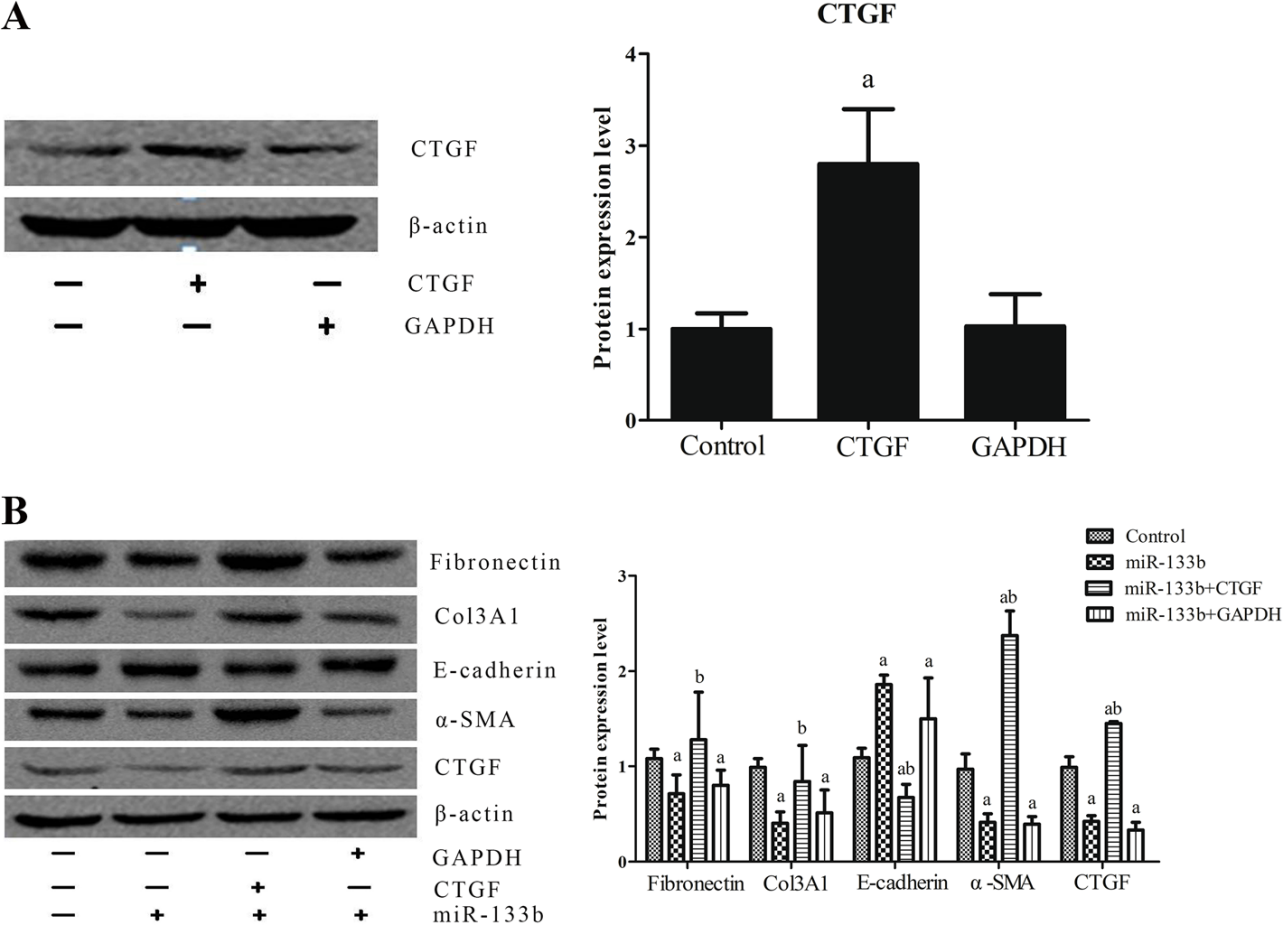
miR-133b inhibited EMT of HK2 cells induced by TGF-β1. **A** Western blotting results after transfection of HK2 cells with CTGF for 48 h. **B** Western blot analysis of HK2 cells stimulated with 8 ng/mL of TGF-β1 for 48 h. a: *P* < 0.05 versus the control group; b: *P* < 0.05 versus the miR-133b group

The cells were transfected with *CTGF* for 48 h, and then they were collected and analyzed by western blotting. Compared to HK2 cells transfected with *GAPDH*, cells transfected with *CTGF* showed a significant upregulation in the expression of CTGF protein, thereby confirming the successful transfection of *CTGF* (Fig. 5A). We stimulated HK2 cells with 8 ng/mL of TGF-β1 for 48 h and performed western blotting. We found that the miR-133b overexpression group showed a significant downregulation in the expression of CTGF, α-SMA, fibronectin, and Col3A1 and a significant upregulation in the expression of E-cadherin as compared to that in the control group. The cells overexpressing miR-133b and CTGF showed a significant increase in CTGF expression. CTGF could revert the inhibition of α-SMA, fibronectin, and Col3A1 expression and the increase in E-cadherin expression mediated by miR-133b overexpression. Furthermore, the group overexpressing miR-133b and CTGF showed significantly higher CTGF and α-SMA levels and significantly lower E-cadherin levels than the control group. The group overexpressing miR-133b and GAPDH showed no significant difference in the expression of various proteins as compared to the miR-133b overexpression group (Fig. 5B).

The results of dual-luciferase reporter assay showed that the miR-133b mimic significantly decreased the luciferase activity of miRGLO-Wt-CTGF but had no significant effect on the luciferase activity of miRGLO-Mut-CTGF (Fig. 3D).

### miR-133b inhibits renal interstitial fibrosis and reduces renal function loss in aged mice with UUO

**Fig. 6:**
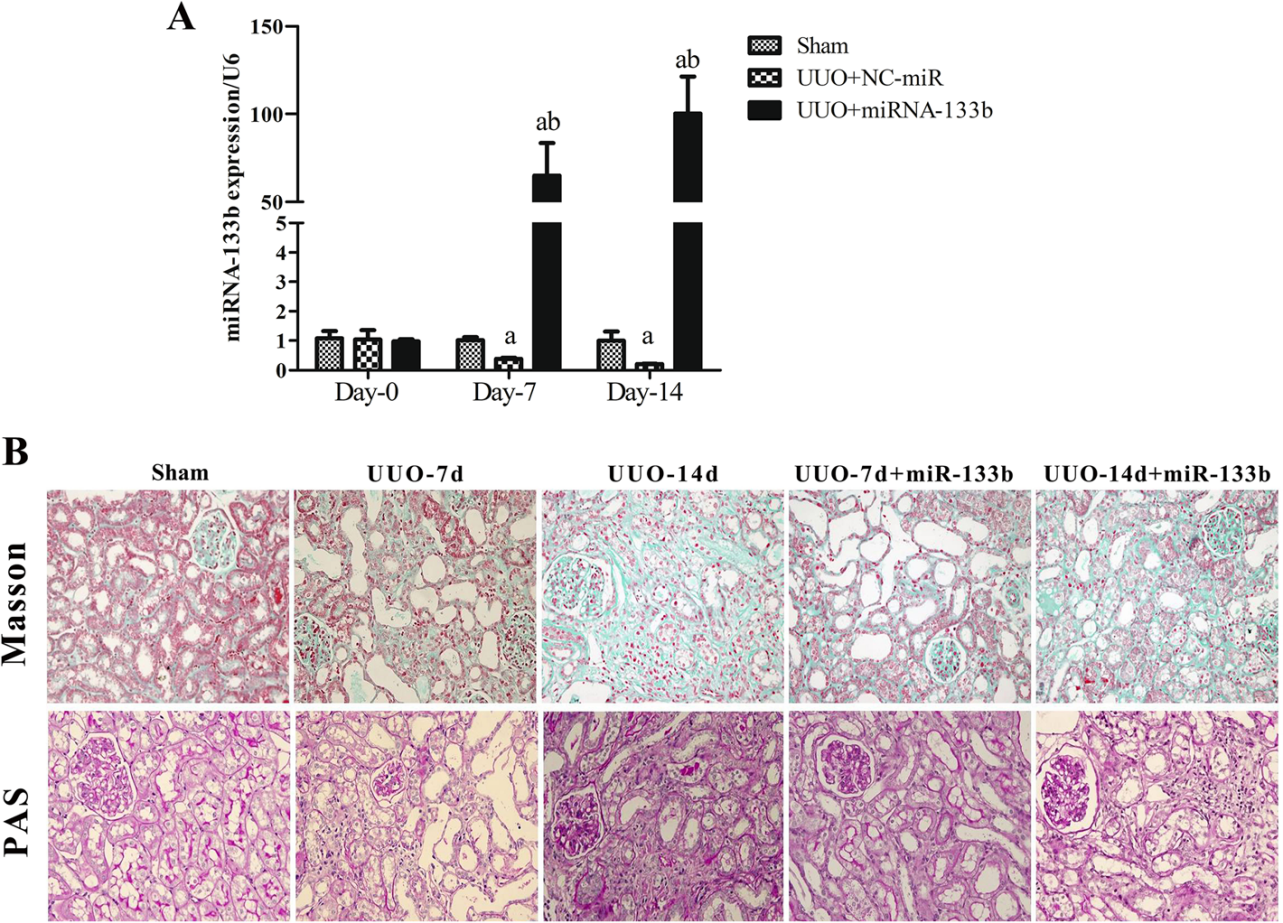
miR-133b inhibited renal interstitial fibrosis in aged mice with UUO. Mice with UUO were administered with an intravenous injection of miR-133b or NC-miRNA transfection complex. **A** Real-time PCR analysis of miRNA-133b expression. **B** Masson’s trichrome and PAS staining of renal tissues as observed under a light microscope (× 100). a: *P* < 0.05 versus the sham group; b: *P* < 0.05 versus UUO+NC-miR group

The miR-133b or NC-miRNA transfection complex was administered to mice (UUO+miR-133b or UUO+NC-miR, respectively) by intravenous injection and the expression of miR-133b was detected by real-time PCR. At 7 and 14 days after UUO establishment, the expression of miR-133b was significantly higher in the UUO+miR-133b group than that in the sham and UUO+NC-miR groups, thereby confirming the successful expression of miRNA-133b. The expression of miRNA-133b in the UUO+NC-miR group was significantly lower than that in the sham group (Fig. 6A).

PAS and Masson’s trichrome staining techniques were performed to observe the morphology of the kidney tissue. Compared to the sham group, the UUO+NC-miR group showed interstitial edema, tubular dilatation, interstitial inflammatory cell infiltration, and a slight increase in collagen levels at 7 days after UUO surgery on the ureter ligation side of the kidney. At 14 days after UUO surgery, the interstitial cell population increased, and collagen expression was significantly upregulated. Compared to the UUO+NC-miR group, the UUO+miR-133b group showed a significant decrease in the level of collagen in the renal interstitium at 7 and 14 days after UUO surgery (Fig. 6B).

We observed the relative area of collagen deposition in the kidney tissue and found it to be significantly higher in all animals with UUO than that in the sham group. The value observed for the UUO+miR-133b group was significantly lower than that obtained for the UUO+NC-miR group at 7 and 14 days after UUO surgery (Table 2).

**Table 2:** Comparison of the relative area of collagen deposition in the kidney tissues of animals from each group

Group	Number	Relative area of collagen deposition (%)
Sham-7d	4	4.10 ± 0.3
Sham-14d	4	4.09 ± 0.3
UUO-7d+NC-miR	5	38.17 ± 5.67*
UUO-14d+NC-miR	5	82.52 ± 7.10*
UUO-7d+miR-133b	5	17.83 ± 4.16*^,#^
UUO-14d+miR-133b	5	40.34 ± 6.18*^,#^

After Masson’s trichrome staining, 10 fields sampled for each animal were observed under a light microscope (× 400), and the area of each field and the area of collagen fibers stained in green were measured by the Image-Pro Plus software. The relative area of collagen deposition was calculated as follows: area of collagen fibers stained in green/area of field of vision × 100%. *P < 0.01 versus the sham group; ^#^*P* < 0.01 versus the UUO+NC-miR group

The results of real-time PCR and western blotting analyses showed that the mRNA and protein levels of CTGF, fibronectin, Col3A1, and α-SMA in the UUO+NC-miR group were significantly higher and that of E-cadherin were significantly lower than the values reported in the sham group. Further, the changes were more significant at 14 days after UUO surgery as compared to that observed 7 days after UUO surgery. At both 7 and 14 days after UUO surgery, the mRNA and protein levels of CTGF, fibronectin, Col3A1, and α-SMA significantly decreased and that of E-cadherin significantly increased in the UUO+miR-133b group as compared to the values observed for the UUO+NC-miR group. However, the mRNA and protein levels of CTGF, fibronectin, Col3A1, and α-SMA in the UUO+miR-133b group continued to be significantly higher and those of E-cadherin were significantly lower compared to the values for the sham group.

The results of the renal function test showed no significant difference in blood urea nitrogen (BUN) and serum creatinine (sCr) levels between the three groups at day 0. At day 7 following UUO surgery, BUN and sCr levels significantly increased in mice with UUO as compared to that in sham mice. The BUN level in the UUO+miR-133b group was significantly lower than that in the UUO+NC-miR group. At 14 days after UUO surgery, BUN and sCr levels in UUO mice were significantly higher than those in the sham mice and the levels in the UUO+miR-133b group were significantly lower than the values reported for the UUO+NC-miR group (Table 3).

**Table 3:** Comparison of renal functions among different groups of animals

Groups	0 days			7 days			14 days		
	*n*	BUN (mmol/L)	sCr (μmol/L)	*n*	BUN (mmol/L)	sCr (μmol/L)	*n*	BUN (mmol/L)	sCr (μmol/L)
Sham	8	6.9 ± 1.0	17.4 ± 0.7	4	6.7 ± 1.0	17.2 ± 0.7	4	7.0 ± 1.4	17.3 ± 1.0
UUO+NC-miR	10	6.9 ± 1.0	17.7 ± 2.1	5	13.8 ± 1.7*	22.5 ± 1.2*	5	18.3 ± 1.5*	28.4 ± 0.7*
UUO+miR-133b	10	6.7 ± 0.6	17.1 ± 1.9	5	10.3 ± 1.1*^,#^	20.5 ± 1.7*	5	14.1 ± 1.3*^,#^	23.2 ± 1.5*^,#^

*BUN* blood urea nitrogen, *sCr* serum creatinine; *P < 0.05 versus the sham group; ^#^*P* < 0.05 versus the UUO+NC-miR group

## Discussion

Tubulointerstitial fibrosis is a chronic and progressive process which affects the kidney tissue during aging as well as in chronic kidney disease regardless of the underlying cause [12]. Kidney fibrosis is characterized by EMT of tubular epithelial cells [13] which contributes to both the destruction of the tubular epithelial compartment and the accumulation of interstitial fibroblasts [14]. TGF-β signaling is thought to play a predominant role in this process [15]. CTGF is a direct downstream early response factor of TGF-β, also known to potentiate TGF-β signaling by directly binding to TGF-β1 through its CR domain [16].

Kidney aging is the basis for susceptibility and high incidence of kidney disease among the elderly. Renal interstitial fibrosis is an important pathological feature of kidney aging, and it is also the main pathogenic event which results in the progression of acute kidney injury to chronic kidney disease [17, 18]. In our previous study, we found that miR-133b-3p was underexpressed in MSC-EVs of aged rats; however, miR-133b can inhibit the epithelial-mesenchymal transition (EMT) of renal tubular cells induced by transforming growth factor (TGF)-β1 [6]. We used a heterogeneous conjoined animal model and found that a younger blood environment could improve renal interstitial fibrosis in aging kidneys [19]. Therefore, we hypothesized that exosomes of bone marrow-derived mesenchymal stem cells might act as a humoral factor to affect renal interstitial fibrosis in the elderly. miRNA-133b can improve renal interstitial fibrosis in the elderly. The low expression of miRNA-133b in stem cell exosomes may be a cause of renal interstitial fibrosis in the elderly. Based on this hypothesis, we conducted this study.

The miR-133b miRNA was first experimentally characterized in mice, and its homologs were identified in several other species, including invertebrates such as the fruit fly, *Drosophila melanogaster* [20]. In the human genome, miR-133 genes include miR-133a-1, miR-133a-2, and miR-133b located on chromosomes 18, 20, and 6, respectively [21]. miR-133 is necessary for the proper development and function of skeletal and cardiac muscles, and its aberrant expression has been linked to many diseases associated with skeletal and cardiac muscles. It is identified as a key factor in cancer development [20–23] and is also known to alleviate cardiac fibrosis in many animal models [8–10]. Lentiviral transfection of miR-133b was found to reduce renal interstitial fibrosis in aged mice with UUO [7] and renal fibrosis in diabetic rats [24]. In the present study, we induced high expression of miR-133b in the kidney tissue through intravenous injection of a miR-133b transfection complex. It was found that miR-133b downregulated the mRNA and protein expression of CTGF, fibronectin, Col3A1, and α-SMA and upregulated the mRNA and protein levels of E-cadherin, thereby significantly alleviating renal interstitial fibrosis and reducing loss of renal function in aged mice with UUO. Thus, we reconfirmed the effect of miR-133b on geriatric renal interstitial fibrosis.

The effect of miR-133b on EMT induced by TGF-β1 is controversial. miR-133b was previously shown to inhibit EMT of HK2 cells induced by TGF-β1 [6, 7]. TGF-β1 downregulated the expression of miR-133a/b in bladder smooth muscle epithelial cells in a concentration-dependent manner, and transfection with miR-133 mimics resulted in the attenuation of TGF-β1-induced expression of α-SMA, extracellular matrix subtype proteins, and growth factors associated with fibrosis [11]. Treatment of primary murine and human hepatic stellate cells with TGF-β1 resulted in significant downregulation in the expression of miR-133a. On the other hand, overexpression of miR-133a in primary murine hepatic stellate cells was shown to decrease the expression of collagen [25]. However, miR-133b was found to be overexpressed in TGF-β1-treated HK2 cells, and miR-133b inhibition attenuated TGF-β1-induced EMT of HK2 cells [24]. Our experiments confirmed the TGF-β1-mediated downregulation of miR-133 expression in a concentration- and time-dependent manner and showed that overexpression of miR-133b significantly inhibited the downregulation of mRNA and protein expression of E-cadherin as well as the upregulation of mRNA and protein levels of α-SMA, fibronectin, Col3A1, and CTGF induced by TGF-β1. The inhibitory effect of miR-133b on EMT of HK2 cells induced by TGF-β1 was further clarified.

Only few studies have evaluated the effect of target genes of miR-133b on the alleviation of tissue fibrosis. A single study on renal fibrosis confirmed Sirtuin-1 as a target of miR-133b in HK2 cells and showed that the inhibition of miR-133b expression resulted in the attenuation of TGF-β1-induced EMT and renal fibrosis through the upregulation of Sirtuin-1 expression [24]. Further, CTGF was found to be a direct target of miR-133 during EMT of cardiomyocytes [9] and bladder smooth muscle epithelial cells [11]. The overexpression of miR-133b significantly downregulated the mRNA and protein levels of CTGF, and CTGF overexpression could reverse the inhibitory effect of miR-133b on TGF-β1-induced EMT of HK2 cells. Moreover, the results of the dual-luciferase reporter assay confirmed that CTGF was a direct target of miR-133b. Therefore, our study confirms for the first time that CTGF is a target of miR-133b and is involved in amelioration of renal fibrosis.

CTGF not only potentiates TGF-β signaling by directly binding to TGF-β1, but also modifies various growth factors and cytokines. Each domain of CTGF can bind to multiple ligands, including insulin-like growth factor-1, fibronectin, bone morphogenetic factors, α5β3 integrin, low-density lipoprotein receptor-related protein 1, vascular endothelial growth factor (VEGF), Wnt, integrins, heparan sulfate proteoglycan, receptor-related proteins, and epidermal growth factor receptor [25]. These cytokines can participate in kidney aging and renal fibrosis through various signaling pathways. Therefore, miR-133b may play different biological roles by downregulating the expression of CTGF and by influencing the processes of kidney aging and renal fibrosis. These effects need to be confirmed through future studies.

## Conclusions

We showed that *CTGF* was a target gene of miR-133b and was involved in ameliorating renal fibrosis. Further, we clarified that miR-133b-mediated inhibition of EMT of HK2 cells induced by TGF-β1 resulted in the alleviation of renal interstitial fibrosis in aged mice with UUO. Improvement of renal interstitial fibrosis in the elderly can help delay kidney aging. These observations serve as a basic research evidence for the development of new drugs based on miR-133b for the amelioration of kidney aging and renal interstitial fibrosis.

## Supplementary Information

**Additional file 1.**

## Data Availability

The datasets used and/or analyzed during the current study will be available from the corresponding author upon reasonable request.

## Abbreviations

miRNA: MicroRNA
MSCs: Mesenchymal stem cells
EVs: Extracellular vesicles
MSC-EVs: MSC-derived extracellular vesicles
TGF-β1: Transforming growth factor-β1
EMT: Epithelial-mesenchymal transition
HK2: Human renal proximal tubular epithelial
ɑ-SMA: Alpha-smooth muscle actin
COL3A1: Alpha 1 chain of type III collagen
UUO: Unilateral ureteral obstruction
CTGF: Connective tissue growth factor
NC-miRNA: Negative control microRNA
FITC: Fluorescein isothiocyanate
PAS staining: Periodic acid-Schiff staining
BUN: Blood urea nitrogen
sCr: Serum creatinine
