# Supplementary material for: Regulation of connective tissue growth factor expression by miR-133b for the treatment of renal interstitial fibrosis in aged mice with unilateral ureteral obstruction
Source: Stem Cell Res Ther. 2021 Mar 10;12:171. doi: 10.1186/s13287-021-02210-2 (PMC7944614; doi:10.1186/s13287-021-02210-2)
Supplement: Supplementary file 1 — Additional file 1. [file 13287_2021_2210_MOESM1_ESM.docx]

The following primers were used:

hsa-miR-133b 5’TTTGGTCCCCTTCAACCAGCTA 3’;

mmu-miR-133b 5’ TTTGGTCCCCTTCAACCAGCTA 3’;

hsa-CTGF 5’ GGCCTCTTCTGTGACTTCG 3 ’and 5’ ATGCAGGGAGCACCATCT 3’;

hsa-α-SMA 5’ ACTGCCGCATCCTCATCC 3’ and 5’CCCATCAGGCAACTCGTAA 3’;

hsa-Ecad 5’ CTGAGAACGAGGCTAACG 3’ and 5’GTCCACCATCATCATTCAATAT 3’;

hsa-FN 5’ GTGCCACCTACAACATCA 3’ and 5’CCACGGTAACAACCTCTT 3’;

hsa-Col3A1 5’ CTTCTCGCTCTGCTTCAT 3’ and 5’CTATCCGCATAGGACTGAC 3’;

hsa-GAPDH 5’ AGCCACATCGCTCAGACA 3’ and 5’CCCAATACGACCAAATCC 3’;

mmu-CTGF 5’ CAAATCCCTGTTGGTGAA 3 ’and 5’TAGGAATCGGACCTTACC 3’;

mmu-αSMA 5’ CAGGGAGTAATGGTTGGA 3’ and 5’GATGATGCCGTGTTCTAT 3’;

mmu-Ecad 5’ GCCAAGCAGCAATACATC 3’ and 5’AAAGGGTTCCTCGTTCTC 3’;

mmu-FN 5’ GCCGAATGTAGATGAGGA 3’ and 5’ATGAGGATAGAGGTGGTAGTC 3’;

mmu-Col3A1 5’ CCCACAGCCTTCTACACCT 3’ and 5’CCAGGGTCACCATTTCTC 3’;

mmu-GAPDH 5’ GACAGCCGCATCTTCTTG 3’ and 5’ACACCGACCTTCACCATT 3’.
